# Dynamics of soil nitrogen availability following conversion of natural forests to various coffee cropping systems in northern Thailand

**DOI:** 10.1016/j.heliyon.2023.e22988

**Published:** 2023-11-28

**Authors:** Phonlawat Soilueang, Kittipong Jaikrasen, Yupa Chromkaew, Sureerat Buachun, Narit Yimyam, Wiriya Sanjunthong, Sasiprapa Kullachonphuri, Suwimon Wicharuck, Nipon Mawan, Nuttapon Khongdee

**Affiliations:** a Department of Plant and Soil Science, Faculty of Agriculture, Chiang Mai University, Chiang Mai, 50200, Thailand; b Department of Highland Agriculture and Natural Resources, Faculty of Agriculture, Chiang Mai University, Chiang Mai, 50200, Thailand; c Faculty of Sciences and Agricultural Technology, Rajamangala University of Technology Lanna Phitsanulok Campus, Phitsanulok, 65000, Thailand; d Energy Technology for Environment Research Center, Chiang Mai University, Chiang Mai, 50200, Thailand

**Keywords:** Coffee agroforestry, Land-use change, Highland agriculture, Nitrogen transformation

## Abstract

Land conversion critically affects soil physiochemical and biological properties, yet very little remains clear about the impact of forest conversion on the N pool and related microbial N transformations. Therefore, this study aimed to examine the dynamics of soil N availability following forest conversion into the different coffee cropping systems, and explore the mechanisms behind these dynamics from the microbial N transformation. Disturbed soil samples from two depths (0–20 and 20–40 cm) were collected from four land uses consisting of three different coffee cropping systems (coffee monocultures (C), coffee agroforestry (FC), coffee associated with persimmon (*Diospyros kaki* L.) (CH)) converted from natural forest and adjacent natural forest (F) in northern Thailand. The soil labile N pools (including ammonium (NH_4_^+^), nitrate (NO_3_^−^), inorganic N (IN), dissolved organic N (DON) contents and microbial biomass N (MBN)) were measured, as well as the soil total N (STN) content. Soil N transformation rates, including net N mineralization, nitrification, and immobilization, were determined using a laboratory incubation experiment. The results showed that the forest conversion to coffee agroforestry significantly increased soil N content by 39.83 % in topsoil, but no significant difference was observed in C and CH soils as compared to F soil (p ≤ 0.05). The three labile N forms (NH_4_^+^, NO_3_^−^ and DON content) were significantly higher under the C, FC and CH soils in both depths, while the coffee monoculture decreased the MBN content. The increases in soil IN, IN/DON and NO_3_^−^/NH_4_^+^ ratios used as an N availability indicator were positively associated with an increase in the N mineralization and nitrification processes following the forest conversion. Interestingly, the N immobilization processes in the F and FC soils were significantly higher than those in the C and CH soils, which indirectly regulated a decreased nitrification rate in F and FC soils in our study. With the exception of the FC soil, the nitrification/N immobilization ratios in the C (4.95) and CH (4.08) soils were higher than those in the F (0.70) soil, indicating an increased N loss risk after forest conversion. Therefore, coffee agroforestry systems have the potential to be effective management strategies for improving soil nitrogen sequestration following forest conversion.

## 1 Introduction

Land-use change (LUC) can have a significant effect on the physical-chemical and biological properties of soil [1,2]. In recent decades, the conversion of forests into intensive agricultural land have been driven by human needs for food, fiber, paper and fuel, among other essentials [3]. It is widely recognized that deforestation occurs mostly in tropical zones due to high food demand [4], with agricultural expansion considered the primary driver responsible for 70–95 % of forest loss [5,6]. Intensive management practices, such as fertilization, understory vegetation control and deep ploughing, are commonly used to enhance plantation growth after changes-land use [7]; Mäkipää et al., 2023). Additionally, the modification of plant species that occurs after forest conversion can impact the soil microbial communities through alterations in litter production and composition, rhizodeposition and microclimate conditions [8,9]. Several investigations have shown that these practices have either positive or negative impacts on modifying soil pH, nutrient levels and the composition of microbial biomass and communities [10,11]. Hence, the rapid expansion of agriculture, specifically the conversion of natural forests into agricultural areas, has emerged as a global concern. Investigating the impacts of land-use change and subsequent management practices on soil nutrient pools, as well as the forms of these nutrients and the associated microbial activities, holds great significance [[12], [13], [14]].

Nitrogen (N) is an essential element in the soils of terrestrial ecosystems, and it has a significant impact on plant development, productivity, and soil fertility [15,16]. Inorganic nitrogen (IN=NH_4_^+^ + NO_3_^−^) and certain organic N compounds that are free and dissolved in soil water or are complexed with soil minerals, including low-molecular-weight compounds like amino acids and urea, are bioavailable forms of N that can limit biomass production in both forestry and agriculture [17,18]. The IN plays a crucial role in plant absorption, highlighting the significance of the supply of IN in influencing plant utilization [19]. In croplands, synthetic N, mainly obtained from chemical fertilizers, serves as the primary N source. On the other hand, in forests, the primary soil N source is the decomposition of organic matter [20]. In contrast to ammonium (NH_4_^+^), NO_3_^−^ form is quickly lost by leaching into groundwater and gaseous emissions [21]. Therefore, the NO_3_^−^/NH_4_^+^ ratio has been utilized as an indicator for assessing soil N availability and N status in various systems. When the NO_3_^−^/NH_4_^+^ ratio is greater than 1, it suggests an open nitrogen cycle [22], indicating high levels of NO_3_^−^ leaching and gaseous N loss [23]. Typically, NI availability is simultaneously controlled by N supply (i.e., via mineralization and autotrophic and heterotrophic nitrification) and consumption (NH_4_^+^ and NO_3_ − immobilization), which can be influenced by both plant cover [24] and land use [25]. Through the process of N mineralization, organic matter converts into NH_4_^+^ [26], which is then oxidized to NO_3_^−^ through autotrophic nitrification [27]. While the primary consumption of IN occurs via microbial NH_4_^+^ and NO_3_^−^ immobilization, plant uptake, denitrification, and leaching [28,29]. Studying overall nitrogen (N) transformations after land use changes helps us understand NH_4_^+^ and NO_3_^−^ soil patterns [[30], [31], [32]]. Typically, higher mineralization and nitrification rates lead to an increase in inorganic nitrogen (IN). It is unclear if these changes in microbial N transformation relate to soil microbial biomass or N pools. Therefore, research on the impact of land use on soil N cycles and microbial processes is growing.

Tropical forests, covering approximately 50 % of known species and an abundance of undiscovered ones, are recognized as one of the most diverse terrestrial ecosystems on our planet (Franca et al., 2020). During the first two decades of the 21st century, Thailand ranked 13th among countries in terms of tropical forest loss, with an estimated clearance of 1,902,664 ha, accounting for 2 % of the overall loss. Therefore, deforestation has become a significant challenge in northern Thailand, particularly in high mountainous regions, mainly due to the establishment of community settlements. Currently, global coffee consumption has been increasing at an average rate of over 2 % annually during the last ten years. In a similar pattern, the Thai coffee industry experienced growth from 2016 to 2020. Thailand's yearly average demand for coffee beans stands at nearly 79,000 tons. The cultivation of coffee by hill tribe farmers, introduced as a cash crop, is gaining importance in the northern highland region of Thailand, with Arabica coffee (*Coffea arabica* L.) being the dominant variety. Additionally, Arabica coffee has been considered a viable substitute for opium in efforts to curb shifting cultivation. One emerging practice involves converting natural forests into coffee plantations. While this transition may bring economic benefits, it also raises important ecological questions, particularly regarding soil nutrient dynamics. Numerous research studies have investigated the effects of converting from natural forests to coffee plantation on soil erosion [33], loss of biodiversity [34], reduced soil fertility, and greenhouse gas emissions [35]. However, impact of converting natural forests into coffee plantations on N pools remains unclear. Moreover, previous research has focused on specific management scenarios, like forest conversion to monoculture systems such as rubber plantations [36], moso bamboo plantations [14,37], Prince Rupprecht's larch, and Chinese pine plantations [38], rubber plantations [39] and Chinese fir and moso bamboo plantations [40], leaving the overall consequences of different cropping systems on forest conversion remain unknown. Therefore, the objectives of this study were to (a) investigate the effects of coffee plantations on the total N content and soil N availability variables, including NH_4_^+^ and NO_3_^−^, dissolved organic N (DON), and microbial biomass N (MBN), aiming to understand the general patterns of soil N availability dynamics following forest conversion in northern Thailand, (b) quantify the alteration of soil N transformation (soil net ammonification, nitrification, N mineralization, immobilization rate) following forest conversion, (c) investigate the responses of soil N cycling-related variables to different coffee cropping systems after forest conversion and (d) establish the relationship between soil net N transformation rates and basic soil physicochemical and N availability, identifying the regulating factors that significantly affected soil N availability variables. Because of the difference in land cover (plant species) and coffee cropping systems following forest conversion, we hypothesized that (a) converting natural forest into coffee plantations would alter the soil N content and availability, (b) the change in soil N availability caused by this land-use change is linked to changes in soil N transformation rates and (c) these changes would vary among different coffee cropping systems within the coffee plantation.

## 2 Materials and methods

### 2.1 Site description

**Fig. 1:**
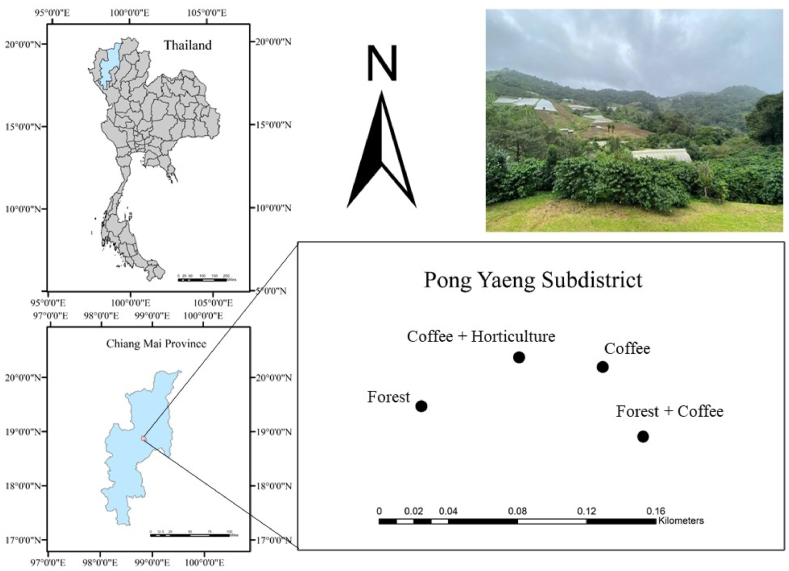
Location of the study sites and the distribution of the field sample sites in the Nhong Hoi Highland Agricultural Research Station in Pong Yeang Sub-district, Mae Rim District, Chiang Mai Province, Thailand.

The study area is located at the Nhong Hoi Highland Agricultural Research Station in Pong Yang Sub-district, Mae Rim District, Chiang Mai Province, Thailand (18°55′19.6″N 98°48′55.0″E) (Fig. 1). The geography of this area is typical of a high hill landscape at an elevation of 850–900 m above sea level, with an average slope of 18–25°. This area is located in a tropical climate with three distinct seasons, which is influenced by Pacific-born typhoons and the Southwest monsoon [41]. The region experiences an annual average precipitation of 1354 mm, mainly occurring between April and August, along with an annual air temperature of 28.5° Celsius. The air temperature fluctuates from 10.3 °C in January to 39.8 °C in July and August. The soils in this area are classified as Alliti-Udic Ferrasols [42].

For comparison purposes, four different land-use types were selected: mixed deciduous forest (F), coffee monoculture (C), coffee agroforestry (FC), and coffee associated with persimmon (*Diospyros Kaki* L.) (CH). These land-use types are located near each other at the bottom of a hillside, approximately 200 m apart. The primary tree species in the mixed deciduous forest include *Bauhinia variegata* L., *Canarium subulatum* Guillaumin, and *Leucaena* sp., with an estimated forest canopy cover of around 70 %. All three coffee cultivation systems were originally established by converting mixed deciduous forests in 1986, and they have been operationed for 36 years. As part of the cultivation practices, the coffee plantations received annual fertilization with urea (210 kg N ha^−1^), superphosphate (50 kg P ha^−1^), and potassium chloride (40 kg K ha^−1^) during late June or early July. Typically, the fertilizer was spread across the soil surface and subsequently plowed to a depth of 0.30–0.35 m. Additionally, as part of the management practice, litterfall was allowed to accumulate on the soil surface each year.

### 2.2 Soil sampling

Soil samples were taken in October 2022, more than a month after chemical fertilizer application, in an effort to reduce the impact of fertilization. At each land-use type, soil samples were collected at five randomly selected points at 0–20 and 20–40 cm soil depths, and these individual samples were subsequently combined, representing 1 replication, totaling 3 replications per land-use type. The soil samples were immediately kept on ice before being taken to a laboratory. Stones and roots were carefully removed by forceps, and the soil was sieved through a 2 mm mesh to eliminate any debris. Some of the soil samples were immediately stored at −20 °C for up to one week for soil microbial community analysis, while another portion was stored at 4 °C for soil ammonification, nitrification and N mineralization rate determination. Additional samples were air-dried and sieved through a 0.15-mm mesh to evaluate the soil's physical and chemical properties. For undisturbed soil sampling, three holes were dug, and then soil samples were collected using intact cores of known volume at different depths, followed by oven-drying at 105 °C for over 24 h to measure soil moisture content and bulk density.

### 2.3 Soil physical-chemical and microbial analysis

The soil pH was measured using a pH meter (Seven Excellence pH meter; Mettler Toledo, OH, USA), with a soil-to-water ratio of 1:2.5 (w:v) after being shaken for 45 min. The contents of soil total nitrogen (TN) were analyzed using an elemental analyzer (CHN–*O*-RAPID, Heraeus, Germany). The quantification of NH_4_^+^ and NO_3_^−^ was detected using the colorimetric method [43] and the modified Cataldo method [44], respectively, after extracting the samples in a 1 mol L^−1^ KCl solution. Microbial biomass nitrogen (MBN) was determined using the chloroform fumigation-extraction method after extracting the samples in a 0.5 mol L^−1^ K_2_SO_4_ solution [45]. The K_2_SO_4_ extracts of the unfumigated samples were considered as dissolved organic N (DON). The total dissolved N content (TDN) was calculated as the sum of TDN and dissolved inorganic N (IN=NH_4_^+^ +NO_3_^−^) contents. To determine the net N mineralization and nitrification rate using microcosm incubation, five sets of 35-mL glass flasks were filled with 5 g of air-dried soil obtained from different land uses. The soil moisture was adjusted to 60 % of the water holding capacity (WHC) using distilled water. Subsequently, the soil was incubated at 25 °C in an incubator for 14 days, and the soil moisture was maintained by watering every 3 days [46]. Three replications were performed per experimental unit. At the beginning and end of the soil incubation, the NH_4_^+^, NO_3_^−^ and MBN contents (mg kg^−1^ soil) were determined as described above. The net ammonification (NAR), nitrification (NNR) and immobilization rate (NIR) were calculated by measuring the difference in NH_4_^+^-N, NO_3_^−^-N and MBN content before and after incubation, respectively. The net N mineralization was assessed by summing the measurements of NAR and NNR.

### 2.4 Statistical analyses

To examine the presence of homogeneous variance and normal distribution, we employed the Shapiro-Wilk test using Statistix 10.0. Subsequently, we conducted one-way analyses of variance (ANOVAs) followed by a least significant difference (LSD) test (p ≤ 0.05) to detect any significant differences in variables across different land uses. The interaction effects of land use type and soil depth on the TN, NH_4_^+^, and NO_3_^−^, DON and MBN were quantified using a two-way ANOVA. Relationships between each soil N variables and soil properties were explored through Pearson correlations in each soil depth. To better visualize the land-use change (LUC), we performed a Principal Components Analysis (PCA) to investigate the relationship among soil N transformation (net ammonification, N mineralization, nitrification, and immobilization rates), soil N availability (NH_4_^+^, NO_3_^−^, NI), and soil properties (pH, EC, SOC, C/N, TN, TC). This analysis was conducted using data collected from the upper soil layers (0–20 cm). All figures were produced using ORIGIN PRO 2021.

## 3 Results

### 3.1 Soil basal physico-chemical properties

The conversion of forest to coffee plantations affected soil physico-chemical properties, and these changes exhibited substantial differences among coffee cropping systems (Table 1). In both soil depths, the highest pH (6.05 and 6.23), EC (103.60 μS cm^−1^ and 58.47 μS cm-1), and TC (33.9 g kg^−1^ and 14.77 g kg^−1^) contents were observed in the FC soil, but the differences were significant only in the topsoil compared to other land use types. In this study, trends in the C/N ratio in soils decreased following forest conversion to coffee monoculture (12.96), coffee agroforestry (12.34), and coffee associated with persimmon (10.39), compared to the forest soil (11.18) in the topsoil. In contrast, in subsoil, the highest soil C/N ratio was found in C soil (14.33), and it was significantly different compared to soils from other land use types.

**Table 1:** Soil properties of different coffee cropping systems and adjacent forest in northern Thailand.

Study site	Soil properties			
	pH	EC (μS cm^−1^)	TC (g kg^−1^)	C/N
0–20 cm				
F*	5.77 b	41.70 b	24.8 b	12.96 a
C	5.83 b	54.23 b	18.4 b	11.18 ab
FC	6.05 a	103.60 a	33.9 a	12.34 ab
CH	5.69 b	45.13 b	17.6 b	10.39 b
20–40 cm				
F	5.69 b	23.60 b	14.70 a	11.14 b
C	5.92 ab	50.07 a	13.63 a	14.33 a
FC	6.23 a	58.47 a	14.77 a	11.39 b
CH	5.67 b	46.87 a	10.74 b	9.52 b

*F: mixed deciduous forest, C: coffee monoculture, FC: coffee agroforestry, CH: coffee associated with persimmon Mean and different letters within a row of values indicate a significant difference (p < 0.05).

### 3.2 Soil total N content and its different pools across various land use types

In this study, the land-use type and soil depths, along with their interactions, significantly altered the TN, DON, and MBN contents (Table 2). However, the interaction between land-use type and soil depth had no significant effects on the NH_4_^+^ and NO_3_^−^ and IN contents. As for soil depths, IN and NH_4_^+^ did not change significantly (p < 0.05), while NO_3_^−^ responded significantly to soil depths.

**Table 2:** Results of mixed-effects analysis of variance on N pools (total N, NH_4_^+^, NO_3_^−^, inorganic N, DON and MBN); F statistic and p value for main and interaction effects. LUT: land use type. In bold: significant (p ≤ 0.05) effects.

Effect	Total N	NH_4_^+^	NO_3_^−^	Inorganic N	DON	MBN
LUT	F = 21.96 (**<0.001**)	F = 33.49 (**<0.001**)	F = 106.64 (**<0.001**)	F = 99.19 (**<0.001**)	F = 29.68 (**<0.001**))	F = 80.6 (**<0.001**)
Soil depth (D)	F = 154.61 (**<0.001**)	F = 1.2 (0.2887)	F = 9.93 (**0.0062**)	F = 3.32 (0.0870)	F = 189.39 (**<0.001**)	F = 366.29 (**<0.001**)
LUT x D	F = 9.35 (**<0.001**)	F = 0.9 (0.4643)	F = 2.92 (0.066)	F = 1.03 (0.4061)	F = 11.36 (**<0.001**)	F = 31.72 (**<0.001**)

The numbers in brackets denote p value.

The total soil nitrogen (TN) content in all land-use types significantly decreased with depth along soil profiles from 0 to 40 cm (Fig. 1a). The highest TN content observed was 2.73 g kg^−1^ under coffee agroforestry (FC) in the topsoil, which was significantly higher than in other land use types (p < 0.05). However, no differences in TN content were observed among the forest (F), coffee monoculture (C), and coffee associated with persimmon (CH) in the topsoil. In the subsoil (20–40 cm), the soil TN content did not show any significant differences among land-use types.

The NH_4_^+^, NO_3_^−^, and inorganic N contents (NH_4_^+^ + NO_3_^−^) under C, FC, and CH soils were significantly higher than those under the forest in both soil depths (Fig. 1d, e, f). When comparing the coffee cropping systems, the NH_4_^+^ content was significantly higher under FC soil compared to F, C, and CH soils in both soil depths. Conversely, the highest NO_3_^−^ content was observed under C soil in both soil depths, and it was significantly higher than the contents under F, C, and CH soils in the topsoil. In the present study, the forest exhibited the lowest NO_3_^−^ content at 4.29 mg kg^−1^ and 4.50 mg kg^−1^ in both topsoil and subsoil, respectively.

The DON contents under C, FC, and CH soils were not significantly different from each other, but they were significantly higher than those under F soil in both soil depths (p < 0.05) (Fig. 1b). Interestingly, FC soil had the highest MBN contents (80.74 mg kg^−1^ and 60.85 mg kg^−1^) in both soil depths, while the lowest MBN content was observed under C soil, and this difference was significant compared to the other land-use types (Fig. 1c).

**Fig. 2:**
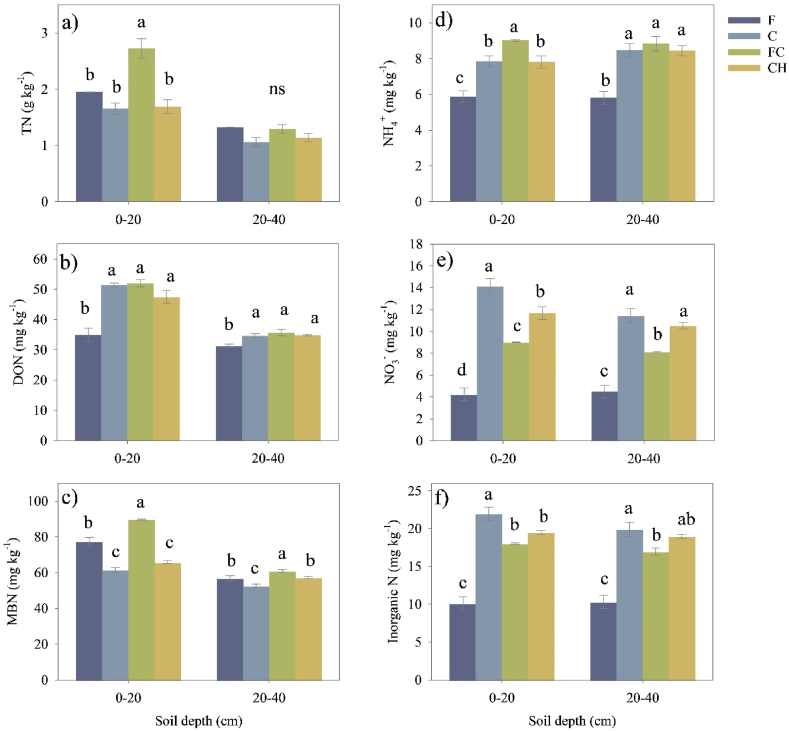
Effects of conversion from mixed deciduous forests to the different coffee cropping systems on the soil (a) total N, (b) dissolved organic N, (c) microbial biomass N, (d) ammonium, (e) nitrate and (f) inorganic N content. The error bars represent the standard error of the mean (n = 3). Different lowercase letters within each panel indicate significant differences between land-use types in each soil layer. These differences were determined using the least significant difference (LSD) test at a significance level of P ≤ 0.05.

The conversion of forests to coffee monoculture (C) and coffee associated with persimmon (CH) significantly increased the NO_3_^−^/NH_4_^+^ ratio and IN (NH_4_^+^ + NO_3_^−^)/DON but decreased the MBN/IN ratio in both soil depths compared to forest soil (Fig. 2a, b, c). However, there were no significant differences in the NO_3_^−^/NH_4_^+^ and IN/DON between F and FC soils in both soil depths.

### 3.3 N transformation rates

In the present study, significantly higher net ammonification (NAR) and mineralization rates (NMR) were observed in F and FC soils compared to C and CH soils. The highest NAR and NMR were found under FC soil (Fig. 5a, c). Additionally, the net nitrification rate (NNR) in C (1.18 mg NO_3_^−^ kg soil^−1^ day^−1^), FC (0.82 mg NO_3_^−^ kg soil^−1^ day^−1^), and CH (1.00 mg NO_3_^−^ kg soil^−1^ day^−1^) soils was significantly higher than that under F soil (0.67 mg NO_3_^−^ kg soil^−1^ day^−1^) (Fig. 5b). When comparing among coffee cropping systems, the highest NNR was exhibited under C soil, which was significantly higher than that under FC and CH soils. In contrast, a higher net immobilization rate (NIR) was found under F (1.02 mg N kg soil^−1^ day^−1^) and FC (0.81 mg N kg soil^−1^ day^−1^) soils (Fig. 5d), resulting in a significantly lower N/I ratio in F (0.70) and FC (1.02) soils compared to C (4.95) and CH (4.09) soils (Fig. 5e).

### 3.4 Relationship between soil physical-chemical properties and soil N availability

The relationship between the physical-chemical variables (i.e., C, TN, SOC, pH, EC, and C/N) and the N availability variables (i.e., NH_4_^+^, NO_3_^−^, IN, DON, IN/DON, and NO_3_^−^/NH_4_^+^) for the soil in each soil depth was examined through Pearson correlation analysis (Fig. 4a and b). MBN showed significant positive correlations with soil C, pH, EC, TN, and C/N, but significant negative correlations with NO_3_ in the topsoil, but not in subsoil. The six types of soil N variables (NH_4_^+^, NO_3_^−^, IN, DON, IN/DON, and NO_3_^−^/NH_4_^+^) exhibited significant positive correlations with each other, but were significantly negatively correlated with MBN/IN in the both soil depths. Principal component analyses (PCA) biplot was used to assess factors that influenced net N transformation rates across different land-use types. PCA showed that net N mineralization and net nitrification were more closely associated with pH (Fig. 6a). There is a strong positive correlation between net ammonification and SOC. However, net immobilization is positively correlated with C/N but negatively correlated with pH. Other soil physicochemical variables (EC, TN, TC) were not closely associated with soil N transformation variables. Additionally, the PCA was also used to determine the relationship between soil N availability and N transformation rate variables (Fig. 6b). The PCA revealed that NAR was significantly and positively correlated with NH_4_^+^, while NNR was significantly and positively correlated with NO_3_^−^ and NO_3_^−^/NH_4_^+^, but negatively correlated with NIR. In this study, the NMR was most strongly correlated with N availability variables, which showed positive correlations with IN, IN/DON content but a negative correlation with MBN/IN. These results indicated that net N mineralization could be employed as an appropriate indicator for determining soil N availability.

## 4 Discussion

### 4.1 Soil total N content and its different pools across various land use types

In this study, the soil TN content and various forms of N could change due to land-use conversion. This study indicated that soil total N content did not decrease at both soil depths following forest conversion into all three coffee cropping systems (Fig. 2a). One possible explanation could be that the application of fertilizers in all coffee cropping systems has led to an increase in N input [47] as the same rate of N loss by the observed increase in N uptake, leaching and mineralization of organic N compounds might be facilitated by the tillage practice employed in coffee plantations [48,49]. According to inorganic N (NI=NH_4_^+^ and NO_3_^−^), our findings indicate that the conversion of mixed deciduous forests into all three coffee cropping systems resulted in higher levels of IN contents (Fig. 2d, e, f). This finding aligns with the observations made by [36]; who reported an increase in NH_4_^+^ and NO_3_^−^ content when converting from a secondary forest to a larch plantation. Additionally, the study conducted by [14] also found a significant increase in IN when converting from a natural evergreen broadleaf forest to a moso bamboo plantation. However, the soil NO_3_^−^ content remained consistently low in all three coffee cropping systems in this study, and this observation could be attributed to various factors. One possible explanation is that the sampling period of this study occurred during the rainy season in our study. Since NO_3_^−^ is an anion that does not easily bind to negatively charged soil colloids, it is more susceptible to be leached [50,51]. Another explanation is that all three coffee cropping systems were cultivated on sloped areas, which easily resulted in the loss of NO_3_^−^ through a surface water flow [52].

In addition, our study observed that converting forests into all coffee cropping systems led to a significant increase in the dissolved organic nitrogen (DON) content (Fig. 2b). This finding is like the study conducted by [53]; who reported an increase in DON content due to the conversion of native forests to Lei bamboo plantations in subtropical China. In contrast [54], reported a significant decline in the soluble organic nitrogen (SON) content in the soil caused by the conversion of natural shrub forests to intensively managed Chinese chestnut plantations. Differences in experimental results were probably due to the implementation of various management practices across different land-use types. In Chinese chestnut plantations, the management practices predominantly included fertilization, tillage and removal of understory species, whereas in Lei bamboo plantations, the focus was on fertilization and mulching [53,55]. Consequently, in our study, a significant increase in DON and TDN content in the soil after forest conversion was primarily attributed to fertilizers and the decomposition of annual mulching materials, such as leaves from trees, coffee, and persimmon. Nevertheless, a decline in the microbial biomass nitrogen (MBN) in the topsoil was found after converting the forests to coffee monoculture and coffee associated with persimmon systems (Fig. 2c). Similar to a previous study conducted by [54] where there was a significant decline in MBN content after 10 years of conversion from shrub forests to Chinese chestnut plantations. The decline in MBN content observed in the coffee plantation can be partially attributed to the lower pH, which is known to inhibit microbial growth [2]. Another possible reason is the significant reduction in soil carbon (C) contents in C and CH soils, which might have had a negative impact on the growth of soil microorganisms [2,56]. This hypothesis is supported by the significant positive correlation observed between MBN and pH in the topsoil (Fig. 4a), as well as 10.13039/100006922TC in our results. Thus, the increase in MBN observed in FC might be attributed to the higher soil pH and TC in FC soil (Table 1).

### 4.2 Changes in soil N availability after forest conversion

**Fig. 3:**
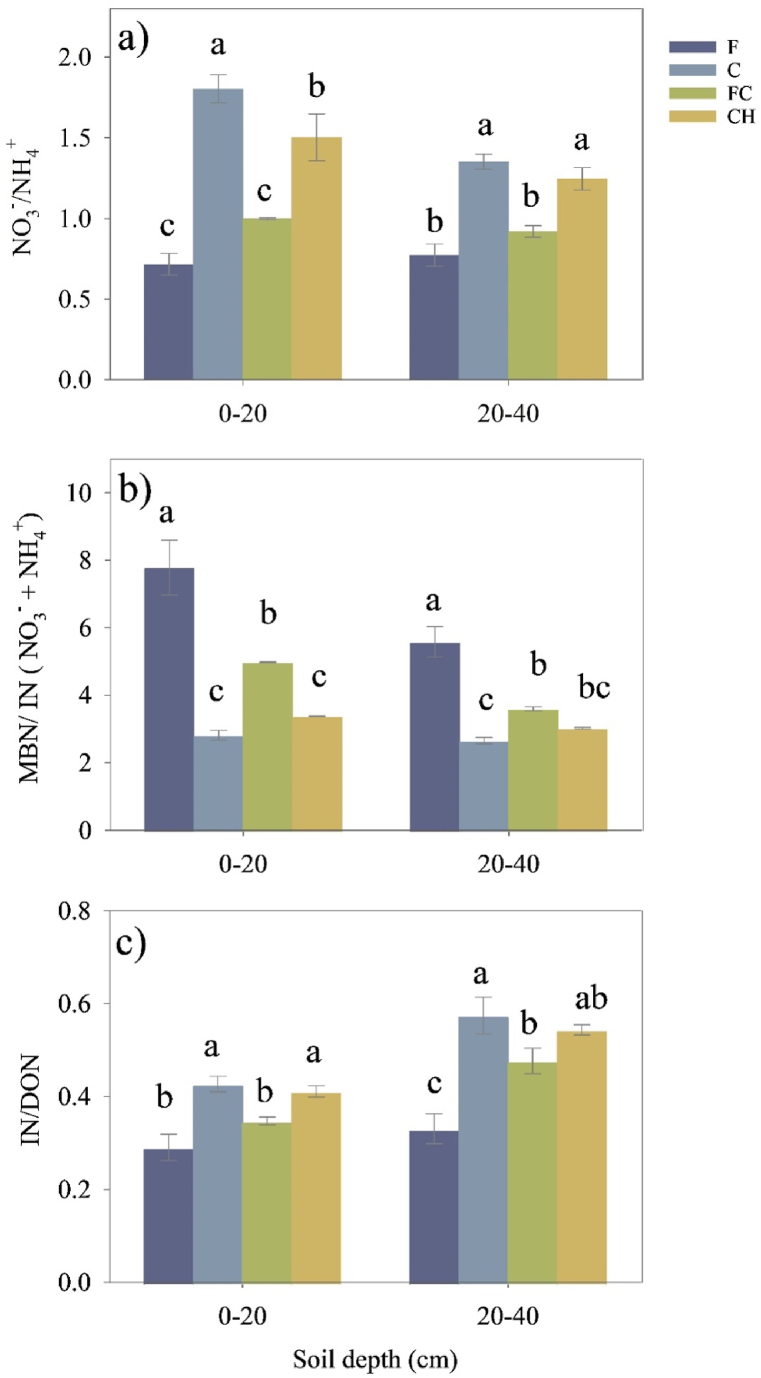
Changes of soil N availability variables including NO_3_^−^/NH_4_^+^ (a), MBN/IN (b) and IN/DON (c) following the conversion of mixed deciduous forests into different coffee cropping systems. The error bars represent the standard error of the mean (n = 3). Different lowercase letters within each panel indicate significant differences between land-use types in each soil layer. These differences were determined using the least significant difference (LSD) test at a significance level of P ≤ 0.05. IN is inorganic nitrogen; DON is dissolved organic nitrogen; MBN is microbial biomass nitrogen; NH_4_^+^ is ammonium; NO_3_^−^ is nitrate. F: mixed deciduous forest t, C: coffee monoculture, FC: coffee agroforestry, CH: coffee associated with persimmon.

The change in the pool size of N availability is determined by the interplay between gross inorganic N production and uptake by plants and microbes. Typically, ratios such as NO_3_^−^/NH_4_^+^, IN/DON, and MBN/IN are used as indicators to represent soil N availability [51,57]. This relationship allows it to serve as a reliable indicator of ecosystem N status, as highlighted in a conceptual model [58]. In N-limited systems, the intense competition between plants and microorganisms restricts the availability of NH_4_^+^ to nitrifiers [59]. Consequently, NH_4_^+^ becomes the predominant form of inorganic N (IN). When the release of NH_4_^+^ through microbial decomposition exceeds the uptake by plants and microorganisms, a larger fraction of NH_4_^+^ is converted into NO_3_^−^ by nitrifiers [58]. As a result, when the NO_3_^−^content exceeds the NH_4_^+^ content in the IN pool (NO_3_^−^/NH_4_^+^ ratio >1.0), it often indicates abundant soil N [60] and an open N cycle [22]. According to our results, the NO_3_^−^/NH_4_^+^ ratios in C, FC, and CH systems exceeded 1.0 (Fig. 3a). This suggests a substantial presence of N in the soil of all three coffee cropping systems, potentially originating from N fertilizer input and the annual incorporation of mulching materials at the study site. However, the NO_3_^−^/NH_4_^+^ ratios in FC soil did not show any significant differences compared to F soil. Therefore, when comparing with FC soil, the higher observed NO_3_^−^/NH_4_^+^ ratios along with lower TN contents in C and CH soils, indicate a greater N loss in the C and CH systems in this study. Typically, NO_3_^−^ is more susceptible to loss compared to other N forms, such as NH_4_^+^, DON and MBN. In the present study, excessive NO_3_^−^ is considered a major contributor to N loss [23], influencing microbial processes and potentially contributing to N_2_O emissions and leaching into groundwater [25,61]. Based on our results, the increase in N availability may be beneficial for plant growth; however, the excessive N availability led to environmental effects through NO_3_^−^ leaching and N_2_O emissions. Therefore, further investigation is required to study the time-dependent changes in soil N availability throughout the growing season and determine if the N supply is adequate to support coffee growth. Additionally, the MBN/IN ratio significantly decreased following the conversion of the forest into all three coffee cropping systems (Fig. 3b). Typically, in cases of IN deficiency, MBN serves as a nitrogen source for the release of IN [62]. The decrease in the MBN/IN ratio indicates that the amount of IN released by microbial activities will exceed the amount consumed by microorganisms. Therefore, the decrease in MBN/IN and increases in NO_3_^−^/NH_4_^+^ ratios indicate a shift from limited IN conditions in the forest to unrestricted N availability after the forest conversion to coffee plantations in this study.

**Fig. 4:**
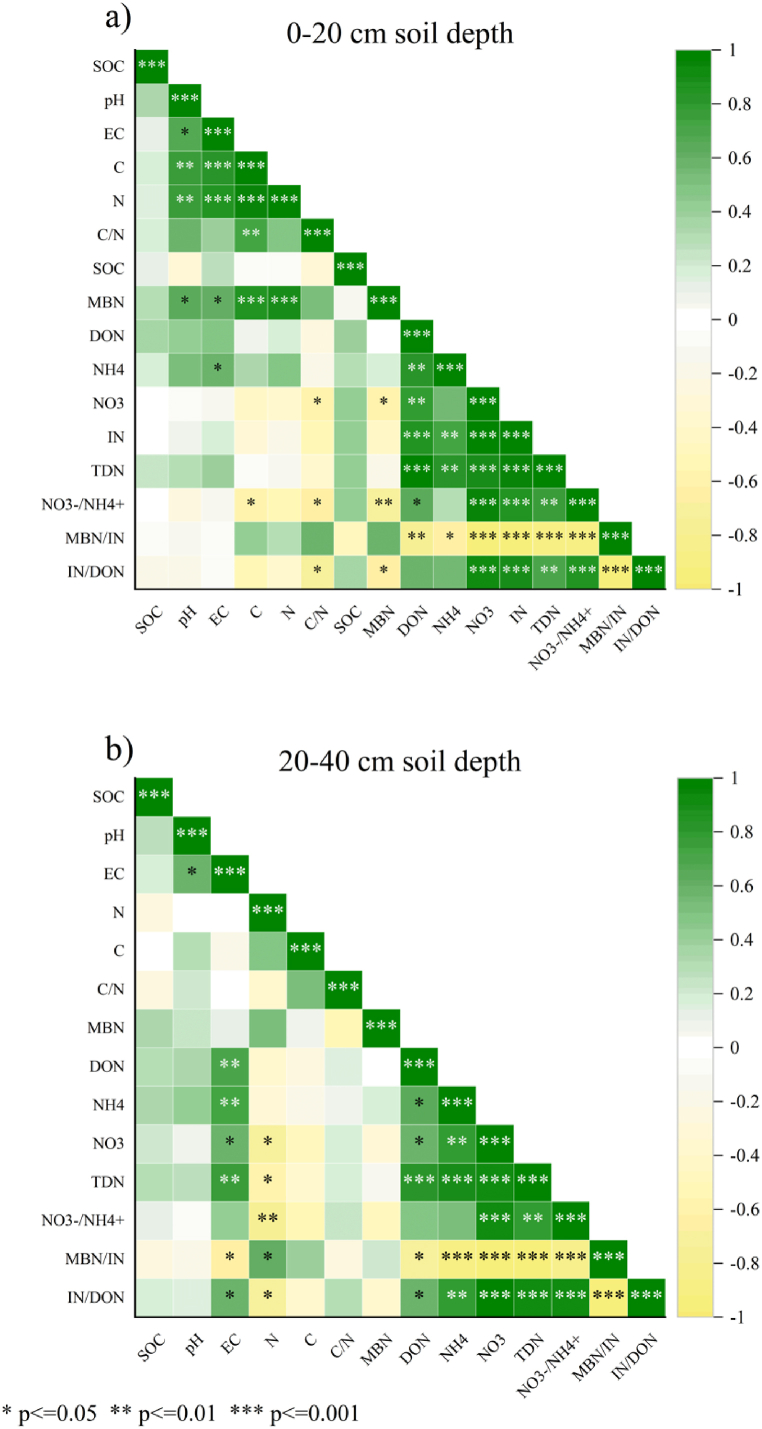
Correlations among soil physicochemical properties and soil N availability in topsoil (a) and subsoil (b). C: Soil total carbon, TN: Total nitrogen, SOC: Soil organic carbon, EC: Electrical Conductivity, pH, C/N: Carbon to nitrogen ratio, NH_4_^+^: Soil ammonium, NO_3_^−^: Soil nitrate, IN: Soil inorganic nitrogen, DON: Dissolved organic nitrogen, MBN: Microbial biomass nitrogen. F: mixed deciduous forest t, C: coffee monoculture, FC: coffee agroforestry, CH: coffee associated with persimmon.

**Fig. 5:**
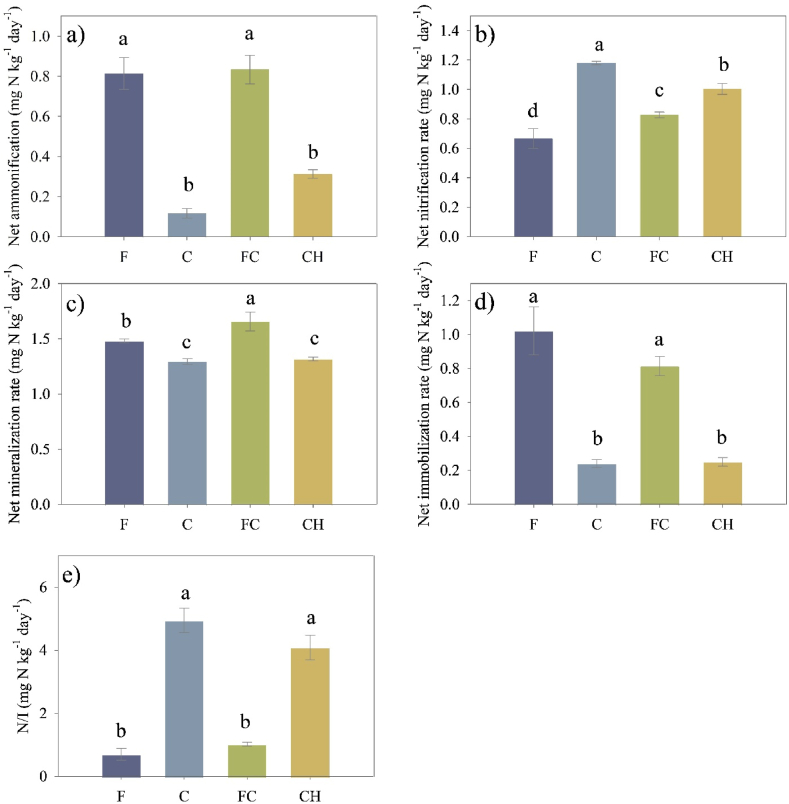
Changes of soil N transformation rates including net ammonification, N mineralization, nitrification, immobilization and N/I following the conversion of mixed deciduous forests into different coffee cropping systems. The error bars represent the standard error of the mean (n = 3). Different lowercase letters within each panel indicate significant differences between land-use types in each soil layer. These differences were determined using the least significant difference (LSD) test at a significance level of P ≤ 0.05. N is nitrification; I is immobilization. F: mixed deciduous forest t, C: coffee monoculture, FC: coffee agroforestry, CH: coffee associated with persimmon.

**Fig. 6:**
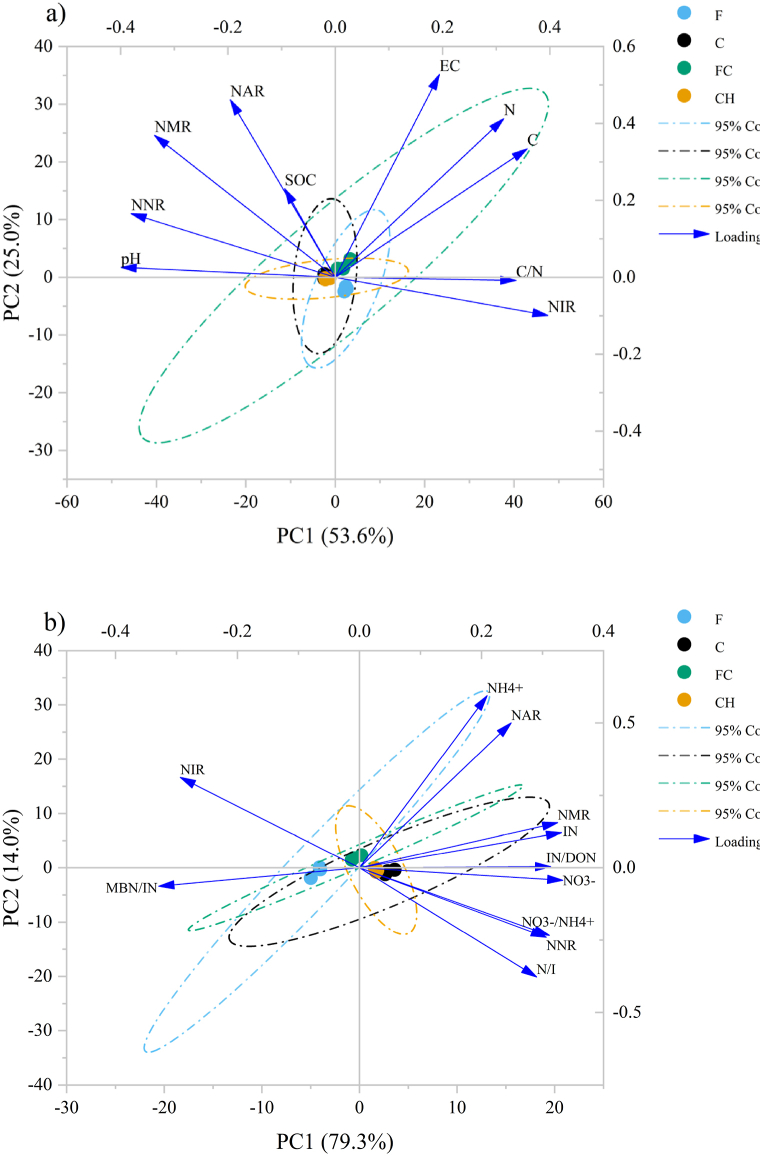
Ordination plots of the results from the principal components analysis (PCA) to identify the relationships among the soil physicochemical properties and N availability and soil N transformation. NH_4_^+^: Soil ammonium, NO_3_^−^: Soil nitrate, IN: Inorganic nitrogen, NAR: net ammonification rate, NIR: net nitrification rate, NMR: net mineralization rate, NIR: net immobilization rate, N/I: nitrification/immobilization. F: mixed deciduous forest, C: coffee monoculture, FC: coffee agroforestry, CH: coffee associated with persimmon.

The IN/DON ratio serves as an alternative measure to assess soil N availability [58]. Typically, an increase in the DIN/DON ratio has often been used to reflect the improvement of soil N availability [58]. In the present study, the increased IN/DON ratio in the C and CH soils indicated an improvement in N availability following the forest conversion, while the FC soil did not show any significant difference compared to the F soil (Fig. 3c). These results show that the FC soil exhibited a lower NO_3_^−^/NH_4_^+^ ratio, IN/DON ratio, and a higher MBN/IN ratio compared to the CH and C soils. These findings could potentially explain the observed increase in soil TN after converting the forest to a coffee agroforestry system, despite the possible decline in N availability. These findings align with the study of [63]; who found that a decreased NO_3_^−^/NH_4_^+^ ratio, IN/DON ratio, and an increased MBN/IN ratio imply increased soil N along with decreased N availability during vegetation restoration in the subtropical China. Interestingly, our study found that coffee production with persimmon and coffee agroforestry had different effects on soil N availability, highlighting the importance of further research into the influence of shaded tree species on N availability.

### 4.3 Microbial N transformation and its relationship to soil N availability

This study hypothesized that microbial N transformation would change after converting a forest into coffee plantations, and this change varied among different coffee cropping systems. In the present study, net N mineralization rate (NMR) increased with the conversion of forest to all coffee cropping systems (Fig. 5c). Our findings are consistent with [64] who found that soil N mineralization increased after converting forest to tea plantations. Based on different coffee cropping systems, the higher NMR was observed in coffee agroforestry (FC) soil compared to coffee monoculture (C) soil. Several studies have reported differences in the rates of N mineralization between agroforestry systems and monoculture agriculture [65,66], while contrasting findings were observed by [67,68]; who did not detect any significant differences. Typically, the N mineralization in terrestrial ecosystems is influenced by various factors, including temperature, soil moisture, and the TN and C/N ratio of the soil [[69], [70], [71], [72]]. However, these factors were not observed or studied in the current study (Fig. 6a). According to the finding of [66]; who revered that the forested land had lower net N mineralization rates compared to the agricultural land, but this did not reflect the actual rate of N transformation. This is because the forested land had higher gross N mineralization rates, which were obscured by the increased gross N immobilization rates [73]. A significantly higher net immobilization in the forest and coffee agroforestry might be one reason for the lowered net N mineralization rate observed in our study, which could explain the absence of a significant relationship found in previous studies. Moreover, the net soil nitrification rate (NNR) showed a significant increase after the conversion of forests into all three coffee cropping systems (Fig. 5b). Among the different coffee cropping systems, coffee agroforestry and coffee associated persimmon demonstrated potential in reducing soil nitrification rates compared to coffee monoculture. The lower soil net nitrification observed in FC and CH soils can be attributed to several reasons. Firstly, as supported by the lowest nitrification rate observed in forest soil, it is possible that plant species in coffee agroforestry systems, which are the same species found in forests, possess the ability to suppress nitrifier bacteria involved in the nitrification process through root exudates. This phenomenon is known as biological nitrification inhibition (BNI) [74]. Meanwhile, the lower nitrification rate in the CH soil compared to the C soil leads us to hypothesize that persimmon plant might also possess BNI abilities. Another contributing factor to consider is the higher net immobilization rate (NIR) in FC soil by heterotrophic microorganisms, which may limit the availability of substrate (NH_4_^+^) for nitrifiers due to the heterotrophs' faster growth rate and stronger affinity for NH_4_^+^ [75]. Consequently, nitrification is indirectly impeded as NH_4_^+^ availability decreases. This phenomenon is commonly referred to as indirect nitrification inhibition, which is in line with the current dispute regarding the ecological explanations behind the observed reduction in net nitrification within specific climax ecosystems, including grasslands and coniferous forests [[76], [77], [78]]. Our study revealed positive correlations between NIR and the C/N ratio, highlighting the significance of these factors in influencing N immobilization (Fig. 6a). However, there was no significant difference in NIR between C and CH soils, but a significant difference in NNR indicated that the lower nitrification observed in the CH soil could be attributed to the phenomenon of BNI. Therefore, it is necessary to assess the potential biological inhibition of nitrifiers by specific compounds associated with certain tree species in both forest and agroforestry coffee systems in future research. According to our study, 10.13039/100013765NI and NO_3_^−^/NH4+ and IN/DON were positively associated with NMR and NNR, respectively (Fig. 6b), which supports the conceptual model [58]; who proposed an increase in both net N mineralization and nitrification as soil N availability improved.

According to [79]; the primary mechanism believed to enhance N retention in forest soils is the process of microbial N immobilization. In the present study, N immobilization significantly decreased after converting the forest into coffee plantations, indicating a high risk of soil N loss. The current study employed the N/I ratio as a measure to determine the primary outcome of ammonium and the potential for NO_3_^−^ leaching losses [80,81]. To minimize N loss through leaching and N_2_O emission, it is advantageous to have lower rates of nitrification relative to immobilization. Our study found that a higher N/I ratio was observed in C soil as compared to FC soil, indicating that coffee monoculture had a higher risk of N loss compared to coffee agroforestry. Our finding thus, one mechanism behind improving N sequestration in coffee agroforestry is to decrease the potential N loss risk by increasing microbial N immobilization along with increased N availability contributed by increased NNR and NMR. Our finding suggests that coffee agroforestry systems could be employed as sustainable coffee farming methods to enhance N recovery after forest conversion and mitigate adverse effects related to N losses.

Nevertheless, the technique of controlled laboratory incubation is widely employed to assess soil N transformation. Nevertheless, it is likely that N mineralization from SOM may be overestimated in laboratory conditions compared to real field scenarios [82]. This indicates that the results from laboratory incubation might not accurately represent the complexities of field conditions, where various biotic and abiotic factors simultaneously impact soil processes. Despite this, only a limited number of studies have ventured into field investigations of N transformation. Hence, further research is necessary to understand N transformation in soils under field conditions, with complementary laboratory incubations being essential to bridge this knowledge gap. Additionally, our study was conducted at only one age of coffee plantation (36 years) following forest conversion, and as a result, this finding may differ from the initial period. Therefore, further research is required to investigate soil N dynamics after forest conversion to coffee plantations at different ages. Furthermore, seasonal changes also play a critical role in soil labile N pools and transformations. Our study, which relied on a single soil sampling, may not fully capture the seasonal effects on soil active N pools after the conversion of a forest to a coffee plantation. Therefore, further investigations are urgently needed to take these factors into account for a more precise identification of the factors that impact soil N availability.

## 5 Conclusion

Our findings clearly show that converting a forest into all coffee cropping systems resulted in a significant increase in NH_4_^+^, NO_3_^−^, IN and DON contents, while there was a decrease in MBN (except in coffee agroforestry) compared to the adjacent forest soil. However, the soil total nitrogen (TN) content did not decrease following the forest conversion into all coffee cropping systems at both soil depths. Overall, all the measured N cycling variables support an increase in soil N availability and a progressive open N cycle after forest conversion into coffee plantations. Additionally, the increased soil N availability was closely associated with the increased N mineralization and nitrification. The conversion from forest to all coffee cropping systems enhanced soil nitrification rates, resulting in a significant increase in susceptibility to soil N loss. However, lower N/I ratios were observed in coffee agroforestry, indicating its potential to reduce the risk of N loss compared to other coffee cropping systems. Overall, N availability improved upon converting the forest into coffee plantations, with variations observed among three different coffee cropping systems, highlighting that coffee agroforestry systems have the potential to be suitable strategies for enhancing soil N recovery after forest conversion and managing coffee plantations sustainably.

## Data availability statement

Data will be made available on request.

## CRediT authorship contribution statement

**Phonlawat Soilueang:** Writing - review & editing, Writing - original draft, Formal analysis, Data curation, Conceptualization. **Kittipong Jaikrasen:** Methodology, Data curation. **Yupa Chromkaew:** Writing - review & editing, Writing - original draft, Supervision, Formal analysis, Conceptualization. **Sureerat Buachun:** Writing - review & editing, Methodology, Formal analysis. **Narit Yimyam:** Writing - review & editing, Resources, Data curation. **Wiriya Sanjunthong:** Writing - review & editing, Project administration, Investigation, Data curation. **Sasiprapa Kullachonphuri:** Writing - review & editing, Formal analysis, Data curation. **Suwimon Wicharuck:** Writing - review & editing, Formal analysis, Data curation. **Nipon Mawan:** Writing - review & editing, Writing - original draft, Supervision, Methodology, Formal analysis, Data curation, Conceptualization. **Nuttapon Khongdee:** Writing - review & editing, Writing - original draft, Visualization, Validation, Supervision, Resources, Methodology, Investigation, Funding acquisition, Formal analysis, Data curation, Conceptualization.

## Declaration of competing interest

The authors declare that they have no known competing financial interests or personal relationships that could have appeared to influence the work reported in this paper.
